# Feeding mastitis milk to organic dairy calves: effect on health and performance during suckling and on udder health at first calving

**DOI:** 10.1186/s12917-014-0267-7

**Published:** 2014-11-25

**Authors:** Katharina Abb-Schwedler, Ariane Maeschli, Renate Boss, Hans U Graber, Adrian Steiner, Peter Klocke

**Affiliations:** Research Institute of Organic Agriculture, Ackerstrasse 21, 5070 Frick, Switzerland; Agroscope Research Station, Schwarzenburgstrasse 161, 3003 Berne, Switzerland; Department of Clinical Veterinary Medicine, Clinic for Farm Animals, Vetsuisse-Faculty, University of Berne, Bremgartenstrasse 109a, 3012 Berne, Switzerland; bovicare GmbH, Hermannswerder Haus 14, 14473 Potsdam, Germany

**Keywords:** Dairy cattle, Heifer mastitis, Feeding milk, *S. aureus*, Calf health

## Abstract

**Background:**

Infection pathways of *S. aureus* udder infections in heifers are still not well understood. One hypothesis is that calves become infected with *S. aureus* via feeding mastitis milk. Especially on small-scale farms, pasteurisers are not economic. The purpose of this randomised comparative study was to investigate the influence of feeding milk containing *S. aureus* genotype B (SAGTB) on the health and development of calves and udder health of the respective heifers. Additionally, a method reducing the bacterial load to obtain safer feeding milk was tested. Thirty-four calves were fed mastitis milk from cows with subclinical SAGTB mastitis. One group was fed untreated milk (UMG). For the other group, milk was thermised at 61°C for one minute (heat treated milk group = HMG). After weaning, calves were followed up until first calving. A milk sample of these heifers was taken at first milking to compare udder health of both groups.

**Results:**

Thermisation of milk led to an effective reduction of *S. aureus* in the feeding milk. 78% of the analysed pools were free of *S. aureus*, a reduction of at least one log was obtained in the other pools.

Quarter milk samples revealed that two heifers had a *S. aureus* intramammary infection, but caused by a genotype different from genotype B.

During the suckling period, the UMG had a significantly higher incidence rate of 1.09 diarrhoea cases per 100 calf days at risk compared to 0.26 cases per 100 calf days in the HMG (p < 0.05).

**Conclusions:**

Under the conditions of this study, no effects of feeding milk containing SAGTB on udder health after first calving were observed. But a power analysis indicated that the sample size in the current setup is insufficient to allow for assessment on mastitis risk after SAGTB exposition, as a minimal number of 4 calves infected (vs. 0 in the HMG) would have shown significant effects. High bacterial load, however, was associated with an increased incidence rate of diarrhoea. Thus, thermisation as a minimal preventive measure before feeding mastitis milk to calves might be beneficial for maintaining calf health.

**Electronic supplementary material:**

The online version of this article (doi:10.1186/s12917-014-0267-7) contains supplementary material, which is available to authorized users.

## Background

Feeding whole milk to dairy calves during the first three months of life is mandatory for organic farms in Switzerland [1]. This feeding regimen is supposed to meet the physiological needs of young calves. Godden et al. [2] found lower morbidity and mortality rates in whole milk-fed calves compared to those receiving milk replacer. Whole milk has a higher energy content and a more balanced composition of nutrients than milk replacer [3]. Maternal hormones and growth factors are included in whole milk but not in milk replacer [4].

Due to economic considerations, milk that is not suitable for sale is frequently fed to dairy calves. This milk often originates from diseased cows and thus is contaminated with antibiotics or pathogenic microorganisms. Selim and Cullor [5] found high concentrations of bacteria, including pathogens, in milk fed to calves. Feeding untreated mastitis milk can facilitate the transmission of infectious pathogens and provoke disease in calves [2].

The mastitis prevalence levels of heifers range from 12.3 to 57% of infected quarters at first parturition [6]. In some studies, environmental mastitis pathogens such as *Escherichia coli* (*E. coli*) and *Streptococcus uberis* (*S. uberis*) have been found to be predominant [7,8], while in others coagulase-negative Staphylococci (CNS) had the highest incidence rate [9,10]. *Staphylococcus aureus* (*S. aureus*) as a major pathogen [11] was also frequently isolated in cases of clinical mastitis around parturition: Waage et al. [12] reported 44.3% of clinically affected heifer quarters infected with *S. aureus*. In a Swedish study, *S. aureus* was reported to be the most commonly isolated udder pathogen in veterinary treated mastitis of primiparous cows [13]. In Switzerland, *S. aureus* genotype B (SAGTB) in particular reaches within-herd prevalence of up to 87.5%, because it is contagious and pathogenic [14].

Epidemiological studies in Switzerland and New Zealand indicated that feeding mastitis milk to calves is a risk factor for heifer mastitis [15,16]. This hypothesis was already tested in different studies with conflicting results [17-20]. Schalm [17] concluded from his trials that udder infections with *Streptococcus agalactiae* (*S. agalactiae*) were transmitted via suckling among pen mates after consuming infectious milk. In another study, milk containing haemolytic Staphylococci was fed to 12 calves and inter-suckling was avoided. Nevertheless, 5 animals suffered from mastitis with haemolytic Staphylococci at calving [18]. Roberson et al. [19] tested herds with a high prevalence of coagulase-positive staphylococcal mastitis and where waste milk was fed to calves for mastitis prevalence of heifers when calving and compared them to low prevalence herds. No statistically significant difference was found. Another study was performed on feeding milk inoculated with *S. aureus.* After first parturition, two out of 29 heifers from the treatment group and 6 out of 35 control heifers were affected with *S. aureus* mastitis [20].

One recommended method to decrease the risk of milk with a high bacterial load for calves is pasteurisation before feeding [21]. It was concluded, however, that this strategy is not economically feasible for small-scale farms. Godden et al. [2] considered pasteurisation unfeasible for less than 23 calves fed per day, while Jamaluddin et al. [22] found it to be unprofitable even for farms with less than 315 calves fed per day. Thermisation is a sub-pasteurisation method that uses temperatures of 57 to 68°C for at least 15 s such that after heating the milk shows a positive reaction to the phosphatase test [23]. With this technique, the number of spoilage bacteria in milk can be reduced markedly with minimal heat damage to milk proteins [24]. Especially for coagulase-positive Staphylococci*,* a time temperature combination of 60°C for 30 s was reported to reduce the bacterial load by 3.3 log colony-forming units (CFU)/ml [25].

The objective of this study was to compare the effect of feeding untreated versus heat-treated whole milk originating from cows affected with subclinical *S. aureus* mastitis on the health of dairy calves during a 3-month suckling period and on their udder health at first calving. An additional objective was to determine the ability of thermisation to reduce the *S. aureus* load in whole milk from cows with subclinical *S. aureus* mastitis. Therefore, a non-blinded randomised comparative study was performed.

## Methods

### Animals and animal keeping

The experimental protocol used in this study was approved by the Cantonal Veterinary Office Aargau (Switzerland) before onset of the study.

A total of 34 calves of 3 breeds (17 Simmental, 16 Holstein, 1 Brown Swiss) born between January and November 2009 were recruited from five different dairy farms (all running free stall housing systems, 40 to 110 cows, within a distance of 10 km from the experimental unit). Four of these calves were twins.

As no data was available concerning the effect size (according to Cohen [26]) of feeding *S. aureus* mastitis milk to calves, it was estimated from the data of an epidemiological study [15]. There, a mean logarithmically transformed Somatic Cell Count (logSCC) of 2.12 was found in first calving heifers from farms practising mastitis milk feeding to calves. Farms not practising this management had a mean logSCC of 1.36. Using WinEpiscope program (WinEpiscope 2.0), assuming a variation coefficient of ≤0.33 corresponding to effect size s ~ 0.7, for two-sided hypothesis-testing, a minimum sample size of 16 animals per group was calculated. In order to have two spare calves available, it was decided to recruit 34 calves.

**Figure 1:**
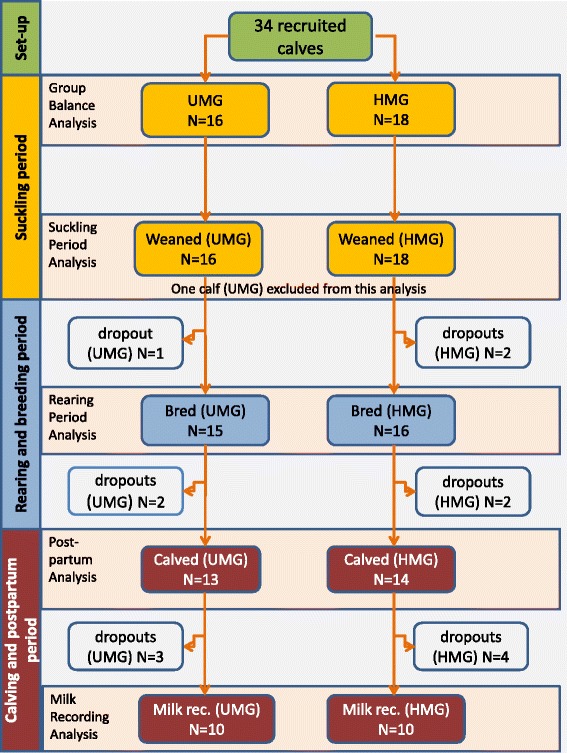
**Study design and animals included in the trial.** Number of animals in different periods and analyses in UMG and HMG, respectively; drop out reasons were death after illness and accidents, slaughtering because of infertility, late calving and lack of milk recording data; UMG = untreated milk group; HMG = heat-treated milk group.

Until introduced into the experiment, the calves were housed in individual calf igloos and fed with the milk of their respective dams that were udder health monitored per monthly SCC-checks and yearly quarter milk sampling for bacteriological culture in the previous and current lactation. In cases of clinical mastitis or elevated SCC (>150,000 cells/ml) additional quarter milk samples were analysed. At the average age of 11 days (SD ±3 days), calves were transferred from their farm of origin to the experimental unit. Calves from every farm were, after initial random selection for the first calf, alternatingly assigned to one of two groups (see Figure 1): 1) Untreated milk group fed with untreated pooled whole milk from cows with subclinical *S. aureus* mastitis (UMG) confirmed by weekly repeated quarter milk sample investigation; 2) Heat-treated milk group fed with heat-treated milk from the same cows (HMG). The groups were housed in two separate small group pens on deep litter straw. These were cleaned twice a day and if necessary fresh straw was substituted. Water was available ad libitum in 10 l buckets and replaced with fresh water twice a day. Hay provided from the farms of origin and concentrates (Kälber-Aufzuchtfutter Proflex Würfel, Alb. Lehmann, Birmenstorf, Switzerland) were also available ad libitum in hayracks and feeders, respectively.

For suckling, two automatic calf-feeders (U40, Urban, Hude-Wüsting, Germany) with two nipples each were installed. Calves were suckled for 12 weeks. The feeding plan of milk available at the automatic feeders is depicted in Table 1. Portions were limited to a maximum of two litres per meal. When calves entered the suckling station, milk was pumped from the storage churns to the feeder unit, heated to 40°C and offered at this temperature for a maximum of 20 minutes. After these 20 minutes, the milk not consumed was discarded. Daily consumable amounts of milk were available for 12 hours during the daytime and were recorded. No milk was available during the night. Calves were stepwise weaned starting in suckling week 7 (see Table 1). Suckling the juvenile mammary glands of pen mates in both groups was documented as far as it was observed during the periods when observers were present (approximately 1.5 hours twice per day).

**Table 1:** **Maximum amounts of milk (litres/calf/day or week, respectively) offered during the suckling period**

**Suckling week**	**Mean amount of milk (l) per calf and day**	**Milk (l) per calf and week**
1	6	42
2	7	49
3	7	49
4	7	49
5	7	49
6	7	49
7	6	42
8	5.8	40.5
9	5.1	35.7
10	4.4	30.8
11	3.4	24.1
12	1.6	11.1
**Maximum milk amount**		471.2

Fed to 34 calves by the automatic feeders during the 12-week suckling period; weaning was performed stepwise starting from suckling week 7.

The internal surfaces of the feeders were cleaned by an automatic programme twice a day. For this purpose, a phosphoric acid-based cleaning agent (Unipred, Timac Agro Swiss, Sion, Switzerland) and hot water were pumped through the milk-conducting parts of the system. There were 12 hours in between the cleaning procedures. In the morning the hoses linking the feeders and the storage churns were also cleaned automatically. The stations were manually cleaned externally daily and the entire equipment twice a week.

### Preparation of milk

The milk used for the pooled feeding milk (PFM) was provided by a farm in 10 km distance, with an evident herd health problem of *S. aureus* mastitis. Untreated cows that were positive for *S. aureus* at culture were considered as PFM contributors for the project; PFM was daily transported to the experimental unit in milk churns immediately after milking. For the UMG, untreated PFM was cooled down to 5°C with an immersion cooler (UK200, Alfa Laval, Lund, Sweden). For the HMG, heat-treated milk was prepared in a commercial 30 l auto-preserving cooker (WAT14, Weck, Wehr, Germany). For this purpose, PFM was heated to 61°C for one minute, filled into 40 l churns and cooled down to 5°C with another immersion cooler. Milk was stored at 5°C in the two 40 l milk churns for a maximum of 24 hours until consumed or discarded, respectively.

### Clinical examination

General health examination and weighing (animal balance, Meier-Brakenberg, Extertal, Germany) were performed upon entering the experimental unit. During the suckling period, the two groups were observed daily, and general clinical examinations were conducted once a week by the first author, including evaluation of the general condition, body temperature (obtained as rectal temperature), the frequency of heartbeat and breath and auscultation of the respiratory tract. Furthermore, the gastrointestinal tract, focussing on appearance and consistency of feces, the central nervous and locomotor systems and the umbilicus were examined. Fever was diagnosed at body temperatures exceeding 39.5°C. Respiratory disease was defined as combination of breath frequency >45, pathological lung sounds and coughing present. Omphalitis was diagnosed in calves with a swollen, painful, warm umbilicus with discharge. Diarrhoea was defined as faeces of runny or watery consistency. During the rearing period, clinical examination was performed once a month, and weight was estimated based on heart girth measurement with a tape (Animeter, Albert Kerbl GmbH, Buchbach, Germany) [27].

If calves showed disease symptoms, the affected animals were individually treated. Treatments were primarily non-antimicrobial following published recommendations so as not to influence the microbial intake [28]. The homeopathic remedy corresponding to the symptoms was searched in the handbook, and 5 globuli of the C30 potency were given to the respective diseased animal twice a day (morning and evening) until the calves recovered. Remedies were adapted after a change of symptoms or if no amelioration of the calves condition was observed within 12 hours after treatment. Only in cases of therapy resistance or life-threatening diseases, conventional therapies with antimicrobials and NSAIDs were undertaken.

### Study design

Both groups were observed during three stages of life: 1) the suckling period; 2) the rearing and breeding period; and 3) the calving and postpartum period. Figure 1 graphically depicts these periods and the involved animals. For the suckling period, calves were kept at the experimental unit. They were housed together with the penmates of their group and fed with the defined milk of their respective group throughout the suckling period. For the analysis of the suckling period, a disease was registered as such only after an individual calf had passed a disease free period of 5 days at the experimental unit, in order to exclude effects from the farms of origin.

After weaning, calves were housed in groups together with calves not participating in the project at their farms of origin. These farms all cooperated with specialised rearing farms located in the mountain regions of Switzerland (1200 to 2300 m above s. l.). Calves were brought there at the age of 4 to 6 months. The first artificial inseminations were performed in winter 2010/2011. The timing of the first insemination depended on individual body-weight, but also according to the alpine pasturing seasons and the farmers’ personal decisions.

Heifers returned for calving to their farms of origin between 5 months and one month prior to the calculated calving dates. Farmers were instructed to contact the first author immediately at the onset of calving symptoms to ensure that the very first milk prior to suckling or milking could be sampled.

### Collection and analysis of milk samples

Of the PFM, daily milk samples were taken immediately after delivery at the experimental unit. Afterwards samples of the heat-treated PFM were taken from the auto-preserving cooker. Both samples were frozen at -18°C for later analysis. Individual composite samples of the PFM-providing cows were weekly taken prior to milking and analysed in a certified routine diagnostic laboratory (Idexx-Diavet, Bäch, Switzerland) to confirm the *S. aureus* shedding in the milk.

PFM samples were analysed at Agroscope research station (Agroscope, Berne, Switzerland) in summer 2012. All PFM samples were investigated in two steps: first the pooled samples were qualitatively analysed using classical culturing methods. In a second step, the number of CFU of *S. aureus* in milk was determined with plate counts. In preparation for the analyses, samples were thawed and pre-warmed in a water bath at 37°C for 10 minutes. Daily samples were merged to weekly pools and plated on 5% sheep blood agar (Biomérieux Suisse, Geneva, Switzerland) and CHROMagar Staph. aureus plates (CHROMagar, Paris, France), specific for *S. aureus* [29]. From heat-treated milk, 100 μl from each pool was plated. From untreated milk samples, only 30 μl was plated on blood agar after the first 10 processed pools grew too densely. All plates were incubated at 37°C. Results were obtained after 24 and 48 hours.

Colonies grown on blood agar were identified according to the guidelines of the National Mastitis Council (NMC) which include morphology, biochemical properties, and detection of haemolysis [30]. *S. aureus*, *S. uberis*, *S. dysgalactiae* and coliforms were referred to as major pathogens, whereas Corynebacterium sp. and CNS as minor pathogens. Concerning the CHROMagar plates, pink coloured colonies were considered to be *S. aureus* and were additionally identified by PCR for the *nuc*-gene [31]. This gene codes for the *S. aureus* specific enzyme thermonuclease. If colonies were *nuc*-positive, they were genotyped according to Fournier et al. [32]. For each PFM sample, one *S. aureus* colony was isolated. In cases, where colonies visually differed from each other, the according number of colonies was isolated.

The quantification of native *S. aureus* in the PFM was performed with serial dilutions. After thawing the pools in a water bath at 37°C for 10 minutes, two 1:10 dilutions with 0.9% sodium chloride solution were produced. Two 50 μl samples of pure milk and one 50 μl sample of each of the two 1:10 dilutions were plated on CHROMagar Staph. aureus plates with a spiral plater (EddyJet, iUL Instruments, Barcelona, Spain). The plates were incubated for 24 hours at 37°C and the colonies were manually counted. If the plates with pure milk were not countable, 1:100 dilutions were cultured and counted again.

Sterile quarter milk samples were taken from the heifers immediately after calving, following the guidelines of NMC [33]. A pre-milking teat disinfectant based on chloraminum (0.5%; Desinficin CL, DeLaval, Lund, Sweden) was applied before sampling. Of each quarter, two 10 ml sterile plastic tubes were filled. Immediately after transport (10 km), the milk samples were frozen at -18°C and stored until analysed. The samples were analysed at Agroscope according to the procedure for the PFM samples, as described above. For each quarter, one blood agar and one CHROMagar Staph. aureus plate was used. Milk was hand plated at 30 μl per plate on blood agar and 100 μl per plate on CHROMagar Staph. aureus by a sterile glass triangle. The plates were incubated at 37°C. Results were obtained after 24 and 48 hours and different pathogens were semi-quantitatively documented. In case of *S. aureus*-suspicious colonies, *nuc*-PCR and genotyping was performed as described above.

SCC and milk yield of the heifers were obtained from the first three routine monthly milk recordings provided by the Swiss breeding associations. Seven heifers (three of the UMG and four of the HMG) were excluded from this data collection, because the respective farm did not participate in the milk recordings.

### Statistical methods

For all periods, variables of both groups were compared using univariate, univariable analysis methods with the exception of the general linear regression modelling that was univariate and multivariable. Group balance was checked in respect to the season of birth, breed, age, weight at arrival and clinical symptoms at arrival at the experimental unit using the Welch Two sample *t*-Test and Fisher’s exact test. Because of unequal variances within both treatment groups, the Welch Two sample *t*-Test was chosen rather than Standard *t*-Test. The disease incidence rates (cases per 100 calf days at risk) and the specific disease incidence ratios with their 95% CIs for diarrhoea and respiratory disease during the suckling period were calculated and comparison between groups was performed using Chi-square-Test. In order to control for carry over effects from the farms of origin, the disease registration period for the analysis started for each calf individually after 5 disease-free days after arriving at the experimental unit. Survival analysis was conducted considering the time from the start of the disease registration period at the experimental unit to the first occurrence of the respective disease (diarrhoea and respiratory disease). Censoring happened at the day of the last feeding at the experimental unit. Survival differences were tested for statistical significance using a log-rank test. The weekly recorded growth rates were compared group-wise by calculating the mean daily growth rate and using the Welch Two Sample *t*-Test.

One calf from the UMG was excluded from the suckling period analysis, but not for further analysis of the post-weaning periods. With the requested five disease free days at the experimental unit before registering diseases, the start day for registering of this calf exceeded the mean start day of registering of the other calves (20^th^ day of life) by 34 additional days. Moreover, this calf was treated with antibiotics.

For the growth and health evaluation during the rearing period, all remaining animals were considered, even if they failed calving afterwards and could not be analysed for calving and udder health parameters (rearing period analysis population in Figure 1). Body weights were only compared to day 440 in the life of every heifer, because that was the earliest age at first breeding. Linear regression modelling was used to model the effects on heifer growth. The mean weight gain per day at the rearing farms was set as the dependent variable. Independent variables were farm of origin, breed, birth season and treatment group as factors and disease days during the suckling period as continuous variable.

Somatic cell count values were transformed logarithmically to logSCC values in order to achieve normal distribution [34]. Comparison of logSCC and milk yield were performed by using the Welch Two Sample *t*-Test.

Significance level in all statistical calculations was set at α = 0.05, statistical trends were indicated in case of α < 0.10. The statistical analyses were calculated with the programme R ver. 2.15.2 base packages and the package epiR for incidence rate comparison [35]. For the post-hoc power analysis the package pwr was used in order to assess the ability of the study to detect prevalence differences in both groups. The function pwr.2p2n.test is based on Cohen’s statistical power analysis methods [26].

## Results

### Effect of heating on bacterial load in milk

In the PFM samples of the untreated milk (41 pools), in all but one of the pooled samples SAGTB was identified. In this single sample *S. aureus* genotype S was detected instead. These results confirmed the cultural findings of the weekly composite samples of the milk-donating cows that were constantly positive for *S. aureus*. Apart from *S. aureus*, growth of CNS, *Streptococcus sp., Corynebacterium sp.* and *Bacillus sp.* was discovered on the plates of the untreated milk pools.

In the heat-treated pools, bacterial growth was seen in all but one pool. Ten of 46 pools (22%) were positive for *S. aureus*. In 7 pools (15%), traces of SAGTB were found. In 3 pools (7%), *S. aureus* genotype S was found. *Corynebacterium sp.* was the most frequently discovered genus in the heat-treated milk; in some pools also CNS, *Bacillus sp.* and *Streptococcus sp.* were detected.

Quantification of *S. aureus* showed a maximum content of 68,050 CFU/ml and a minimum content of 1,330 CFU/ml in untreated milk. The median was 2,468 CFU/ml. In the heat-treated milk, 37 pools were negative for *S. aureus*, the maximum was 1,450 CFU/ml (median 0 CFU/ml). Compared to the corresponding untreated milk samples, there was a one log reduction (90% reduction of *S. aureus* CFU) in the heat-treated milk samples containing *S. aureus.*

## Group balance

Considering the birth season, breed, age at arrival and body-weight at arrival, the two groups were not significantly different from each other at the beginning of the experiment. In contrast, with regard to the health status at arrival at the experimental unit, HMG calves showed clinical symptoms of disease in 9 out of 18 animals (50%), whereas, in the UMG 3 out of 16 calves (19%) showed clinical symptoms, although the difference was not significant (p = 0.08). Diarrhoea was the disease most often recorded (7 HMG calves, 2 UMG calves). With the requested five disease free days before registering diseases after arrival for the suckling period analysis, both groups entered the analysis period with a mean calf age of 20 days (SD ±5 days).

### Suckling period

**Figure 2:**
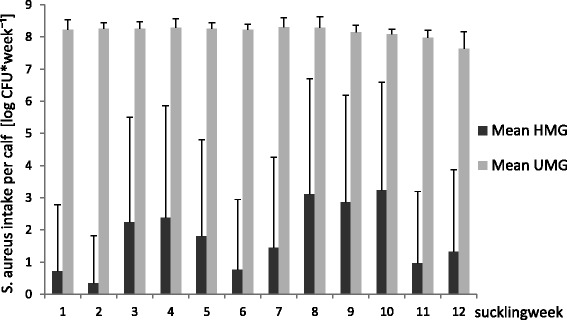
***S. aureus*** **intake during the suckling period.** Mean and SD of total *S. aureus* CFU intake per calf and week during the 12-week suckling period of 34 calves fed untreated (UMG) and heat-treated milk (HMG), respectively; UMG = untreated milk group; HMG = heat-treated milk group; log = log10.

The median (quartile) of the total amount of milk consumed per calf in the trial was 436.3 (422.3-481.8) kg. Figure 2 shows the mean weekly bacterial intake of *S. aureus* CFU during the 12-week suckling period.

Disease incidence rate for diarrhoea in the UMG was 1.09 per 100 calf days at risk; in the HMG, the rate was 0.26 per 100 calf days at risk (p < 0.05). The incidence rate ratio was 4.22 [95% Confidence Interval (CI): 1.01; 24.68]. The survival analysis confirmed the significant differences between the groups concerning diarrhoea cases per day at risk (p < 0.05). While in the HMG the first analysed diarrhoea episodes (n = 3) lasted 2.3 days in mean (SD ±0.6 days), in the UMG (n = 9) they lasted 6.3 days in mean (SD ±3 days). The difference in duration was significant (p = 0.004). In the HMG, there were no second episodes during the suckling period, while in the UMG, there were three relapses, lasting 3.3 days in mean (SD ±1.52 days). The incidence rate for respiratory diseases did not differ significantly between the two groups. The mean daily weight gain during the suckling period was 715 g/day (SD ±9 g/day) in the UMG and 775 g/day (SD ±9 g/day) in the HMG (p = 0.06).

Conventional therapies were administered to two calves of each group. One calf of the UMG had a buccal abscess and severe lesions on the oral mucosa and the tongue. It was treated with Oxytetracyclin (Engemycin®, MSD Animal Health GmbH) 8 mg/kg once a day for four days administered by the intramuscular route and Tolfenamin Acid (Tolfendine®, Vetoquinol AG) 4 mg/kg once per day for two days administered by the subcutaneous route. Another calf of the UMG had a painful bronchitis or/and pleuritis and was treated with Benzylpenicillin (Norocillin®, Arovet AG) 12000 I.U. Penicillin/kg administered by the intramuscular route and Tolfenamid Acid (Tolfedine®, Vetoquinol AG) 2 mg/kg administered by the subcutaneous route both once per day for three days. One calf of the HMG was suspected to have osteomyelitis. It was treated with Benzylpenicillin (Norocillin®, Arovet AG) 12000 I.U. Penicillin/kg for four days administered by the intramuscular route. And the second calf of the HMG suffered from a severe omphalitis and was treated with Amoxicillin (Betamox®, Arovet AG) 15 mg/kg once a day for five days administered by the intramuscular route.

Suckling of pen mates and being suckled by pen mates was observed in all calves of both groups. Calves were observed being suckled on an average of 22% of the observation periods (SD ±8% of the observation periods). They were observed suckling pen mates on an average of 21% of the observation periods (SD ±7% of observation periods) each.

At the end of the suckling period, there were 16 calves in the UMG and 18 calves in the HMG (Figure 1).

### Rearing and breeding period

During the rearing and breeding period, no significant differences in health and performance parameters for both groups were found.

Linear regression modelling showed that differences in weight gain depended on the farm of origin. The results are depicted in Table 2.

**Table 2:** **Results of the linear model performed for the rearing and breeding period**

**Variable**	**Level**	**Coefficient**	**Std. Error**	**T value**	**p**
Intercept		711.3	36.6	19.4	<0.001
Treatment	HMG	Reference			
	UMG	−6.5	41.2	−0.2	0.87
Calf origin	Farm 1	Reference			
	Farm 2	−238.7	55.6	−4.3	<0.001
	Farm 3	33.1	48.4	0.7	0.50
	Farm 4	256.0	85.6	3.0	0.006

Final linear model after removing not significant variables by stepwise backward method (breed, birth season, and disease days during suckling time) for the significant influence factor “farm of origin” on weight gain during the rearing and breeding period; UMG was treated as fixed model factor of investigation.

In this period, 7 heifers were lost. Three of these dropped out prior to breeding, one of the UMG and two of the HMG (Figure 1). The reasons for drop out were: Two heifers were killed in accidents (one of the UMG and one of the HMG) and two after illness (both HMG). Necropsy was performed in the latter two animals. The first one died after a severe episode of diarrhoea and the resulting metabolic disorders. The other heifer had to be euthanised because of a progressive paralysis of the hind limbs, caused by a Waller degeneration of the neurons. Two heifers were infertile and therefore slaughtered (both UMG). One heifer of the UMG was calving late after all the others. It was therefore excluded from the postpartum and milk recording analysis.

### Calving and postpartum period

Out of the initial 34 calves, 13 animals remained in the UMG and 14 in the HMG for the postpartum period analysis (Figure 1).

Examination of the heifer quarter milk samples revealed the growth of *S. aureus* in two animals, one from each group from two different farms. Both heifers showed three affected quarters and both of them carried *S. aureus* genotype C in all of these quarters. Both dams of the two heifers were never tested positive for *S. aureus* mastitis in the lactations before and after giving birth to these calves. Given the sample size considered, significance level of 0.05 and power set to 0.8 and assuming that no infections occurred in the HMG, power analysis indicates a total of 4 calves infected (31%) that would have been necessary to detect a statistically significant effect of feeding SAGTB contaminated milk on homologous SAGTB infection after calving.

**Figure 3:**
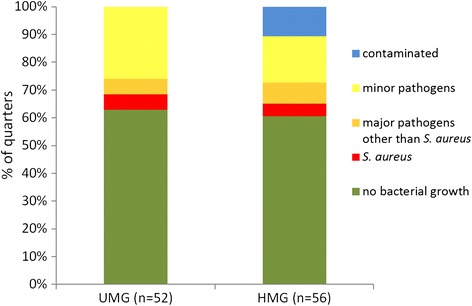
**Results of the bacteriological culturing of heifers’ quarter milk samples.** Samples collected from 27 heifers (108 quarters) immediately after first calving; contaminated means three or more different pathogens per plate.

Thirteen out of 14 heifers (20/56 quarters, 36%) of the HMG and 10 out of 13 heifers (18/52 quarters, 35%) of the UMG were infected with udder pathogens. The types of pathogens involved and their prevalences are depicted in Figure 3. Clinical symptoms of mastitis were not observed.

Results of 10 calves of each group were available for the analysis of the milk recording data (Figure 1). Comparison concerning SCC measurements and milk yield did not reveal any significant difference between the two groups. At first recording, there was a trend (p = 0.09) for higher logSCC values in the HMG (mean 2.5; SD ±1.8) as compared to the UMG (mean 1.2; SD ±1.5).

## Discussion

The udder health data from this trial did not reveal any significant difference between calves fed with heat-treated and untreated mastitis milk. Results from the suckling period, however, indicated that calves fed with unheated mastitis milk suffered more diarrhoea days than calves receiving milk after heat treatment.

Blinding was not possible in this study as the same person prepared the milk and took care of the animals. Housing at the experimental unit was designed to separate the two groups from each other in order to prohibit microbial carryover on calves of the other group.

As we collected the milk sample from each heifer immediately after calving, it seems reasonable to rule out that the pathogens ingested during the suckling period might cause mastitis. Intramammary *S. aureus* infection discovered later might much more likely have been acquired via alternative infection pathways, for example during the milking process or by cow to cow transmission [36]. We intended to exclude these two factors with the chosen study design. In addition, there is evidence from the literature that the majority of intramammary infections occur at the beginning of the lactation [13]. Even subclinical cases should have been detected during laboratory analysis thanks to the high sensitivity of the agar plates [29]. Additionally, 100 μl of milk was plated on a separate plate per quarter to increase sensitivity [37].

Although under the conditions of this study, there was no influence of the quality of the feeding milk on udder health, this finding cannot be generalised, as the focus of the study was exclusively on the transmission of *S. aureus*. Other pathogens present in waste milk, such as *S. agalactiae* might have other transmission pathways and may infect suckling calves [17]. And the milk used for this trial came from one specific farm and contained mainly SAGTB.

The mean number of CFU of *S. aureus* in the feeding milk changed from week to week, according to the individual shedding patterns of the milk-donating cows. Nevertheless, we decided not to use inoculated milk, because we aimed at mimicking field conditions. Other dosages as well as combinations with other microorganisms might have caused other effects. Apart from the study of Barto et al. [20], precise quantities of *S. aureus* fed in milk can hardly be found in the literature.

Because of labour and material intensity the PFM samples were analysed after the analysis of the heifers’ milk samples. Despite the long storage time the analysed quantities should reflect the original *S. aureus* content as it is recorded that freezing does not affect the viability of *S. aureus* in milk samples [38].

Investigations that indicate feeding mastitis milk to calves as being a risk factor for heifer mastitis might reflect an indirect correlation and have to be interpreted carefully. A possible interpretation is that farmers feed mastitis milk to calves due to a serious mastitis problem present in their herd, which represents another risk factor for heifer mastitis [16]. Feeding waste milk to calves could also be an indicator of lower hygiene standards on a respective farm so that the transmission of *S. aureus* is facilitated by an alternative infection pathway as, for example, not wearing gloves at milking or not cleaning milking equipment properly. Such effects were minimized by the particular design chosen in our study, as calves were not raised in herds with an evident mastitis problem.

Some epidemiological studies include the whole range of heifer mastitis pathogens, whereas we put the emphasis on one particular pathogen. Due to the lack of information about heifer SAGTB prevalence in general and, particularly, after exposition with SAGTB during the suckling period, sample size was primarily calculated based on the Cohen’s effect size mentioned by Ivemeyer et al. [15] regarding postpartum SCC difference. Given our setting (considering 17 calves in each group, significance level 0.05, power 80%), we calculated that provided missing SAGTB cases in the HMG, a number of 4 cases (31%) of SAGTB infection in the UMG would detect a statistically significant effect of feeding contaminated milk to suckling calves on SAGTB prevalence in heifers. Results of the presented study suggest that expectable effects of a comparable setting have to be rather small, and with respect to Cohen [26] likely less than 0.2. Given the sample size calculation performed by Cohen’s algorithms, this would require a sample size of nearly 1000 calves for the entire study to detect a difference of prevalences in both groups. Hence, the study results indicate that further research on this topic would have to consider an immense study setting upscaling. Considering the fact that in the current study not a single case of SAGTB was detected after exposition with SAGTB contaminated milk, it has to be evaluated carefully whether this effort is justified at all.

Analysis of the milk samples taken immediately after calving revealed that only two out of 27 heifers had intramammary infection with *S. aureus*. The genotypes, however, were different from those found in the milk during the suckling period. Therefore, it can be ruled out that bacteria from the milk fed in the experiment during the first months of life caused the mastitis of these two heifers in this later period of life. As the dams of these two heifers had never been tested positive for intramammary infections with *S. aureus* in the lactation before and after the birth of both calves, it is very unlikely that they had transmitted *S. aureus* via milk to their calves during the first days after birth.

Heat treatment of the feeding milk was thermisation. In the EU-definition [23] the phosphatase test is recommended to distinguish thermisation from pasteurisation. In this trial, we rather standardised the time-temperature combination to obtain a more exact and repeatable method. The phosphatase test, therefore, was not performed during the trial. By thermisation, inactivation of *S. aureus* was achieved in 78% of the samples. In the other 22% of samples, *S. aureus* CFU were reduced by one log compared to untreated milk. Also, the shift of the bacterial spectrum should be pointed out: in untreated milk, *Staphylococcus sp.* comprised the main part of the bacterial load. After heating, *Corynebacterium sp.* was most frequently found. The reduction of bacteria and the switch of the bacterial spectrum that disadvantaged the major pathogen fauna seem to have had an effect on calf health. On the other hand, the bacterial load was not reduced as effectively as after pasteurisation. Further investigation is needed to measure more accurately the cost effectiveness of thermisation compared to pasteurisation. The obtained reduction of the bacterial load, however, is expected not to be sufficient for some specific bacteria such as *Mycobacterium paratuberculosis* or *Mycoplasma sp.* [39,40]. We did not offer the prepared milk to the calves for longer than 12 hours per day. This was in accordance with the traditional Swiss feeding practice, where calves obtain their milk within this period. The same milk was used for both groups, but heat-treated for one group. Accordingly, the same amount of nutrients was provided to all calves but without the high bacterial load for the group obtaining the thermised milk. Side effects of heat-treating milk, such as the change of protein structure or loss of vitamins can be reduced to a minimum with the method advocated in our study [24].

The lower risk for diarrhoea in calves after ingesting heat-treated milk coincides with Jamaluddin’s findings [41]. Episodes of diarrhoea in the group obtaining pasteurised milk were shorter and thereby less severe. *Staphylococcus aureus* is not a typical diarrhoea-causing pathogen in calves, but its enterotoxins are well known as contributing to immunosuppression which can explain a higher susceptibility to disease [42]. The presence of enterotoxins in milk was not analysed. But the minimal bacterial count of 10^5^ CFU/g, necessary for enterotoxin formation, was reached in the pool milk. And high counts of viable bacteria are considered to play a role in adverse effects in calf health [5]. Some diarrhoea episodes, especially in the UMG, are overlapping with the episodes of other calves in the group (see Additional file 1). As causative organisms were not differentiated, a transmission of enteric pathogens from one calf to another cannot be excluded. Nevertheless, calves of the UMG seem to have been more prone to diarrhoea. During the suckling period, a trend to higher weight gains was observed in the group fed with heat-treated milk, which was also stated in earlier studies [2,41]. An explanation might be that diseases such as diarrhoea can adversely affect growth [43].

## Conclusions

Feeding female calves with milk containing a high load of SAGTB under the conditions of this study did not affect udder health of these animals at first calving. As the power analysis revealed, generalisation of this result however, is not justified. The heat treatment of milk originating from cows suffering from *S. aureus* mastitis is a reasonable measure to reduce the risk of calf diseases, particularly diarrhoea. For small-scale farms without paratuberculosis problems, where a professional pasteuriser is not available, thermisation for one minute to 61°C might be a promising alternative to reduce calf morbidity. Furthermore, this technique is sustainable, as discarding the milk from cows with *S. aureus* mastitis can be avoided.

## Additional file

Additional file 1:
**Data diarrhoea, Calendar diarrhoea, Legend.** “Data diarrhoea” shows the exact individual dates of when each diarrhoea episode of each calf took place. “Calendar diarrhoea” gives a graphic overview of the episodes in a calendar. “Legend” explains the abbreviations and colour used in the previous tables.

## Abbreviations

CFU: Colony-forming units
CI: Confidence interval
CNS: Coagulase-negative staphylococci
*E. coli*: *Escherichia coli*
SAGTB: Genotype B of *S. aureus*
HMG: Heat-treated milk group
logSCC: Logarithmically transformed SCC by the use of log10
NMC: National Mastitis Council
NSAID: Non-steroidal anti-inflammatory drug
PFM: Pooled feeding milk
SCC: Somatic cell count
SD: Standard deviation
*S. aureus*: *Staphylococcus aureus*
*S. agalactiae*: *Streptococcus agalactiae*
*S. dysgalactiae*: *Streptococcus dysgalactiae*
*S. uberis*: *Streptococcus uberis*
UMG: Untreated milk group
